# Circular RNAs: New layer of complexity evading breast cancer heterogeneity

**DOI:** 10.1016/j.ncrna.2022.09.011

**Published:** 2022-10-12

**Authors:** Alyaa Dawoud, Zeina Ihab Zakaria, Hannah Hisham Rashwan, Maria Braoudaki, Rana A. Youness

**Affiliations:** a Molecular Genetics Research Team (MGRT), Pharmaceutical Biology Department, Faculty of Pharmacy and Biotechnology, German University in Cairo, 11835, Cairo, Egypt; b Biochemistry Department, Faculty of Pharmacy and Biotechnology, German University in Cairo, 11835, Cairo, Egypt; c Clinical, Pharmaceutical, and Biological Science Department, School of Life and Medical Sciences, University of Hertfordshire, Hatfield, AL10 9AB, UK; d Biology and Biochemistry Department, School of Life and Medical Sciences, University of Hertfordshire hosted By Global Academic Foundation, New Administrative Capital, 11586, Cairo, Egypt

**Keywords:** CircrRNAs, Breast cancer, Diagnosis, Prognosis, Precision medicine, Heterogeneous tumor, Biomarkers

## Abstract

Advances in high-throughput sequencing techniques and bioinformatic analysis have refuted the “junk” RNA hypothesis that was claimed against non-coding RNAs (ncRNAs). Circular RNAs (circRNAs); a class of single-stranded covalently closed loop RNA molecules have recently emerged as stable epigenetic regulators. Although the exact regulatory role of circRNAs is still to be clarified, it has been proven that circRNAs could exert their functions by interacting with other ncRNAs or proteins in their own physiologically authentic environment, regulating multiple cellular signaling pathways and other classes of ncRNAs. CircRNAs have also been reported to exhibit a tissue-specific expression and have been associated with the malignant transformation process of several hematological and solid malignancies. Along this line of reasoning, this review aims to highlight the importance of circRNAs in Breast Cancer (BC), which is ranked as the most prevalent malignancy among females. Notwithstanding the substantial efforts to develop a suitable anticancer therapeutic regimen against the heterogenous BC, inter- and intra-tumoral heterogeneity have resulted in an arduous challenge for drug development research, which in turn necessitates the investigation of other markers to be therapeutically targeted. Herein, the potential of circRNAs as possible diagnostic and prognostic biomarkers have been highlighted together with their possible application as novel therapeutic targets.

## 1 Introduction

**Fig. 1:**
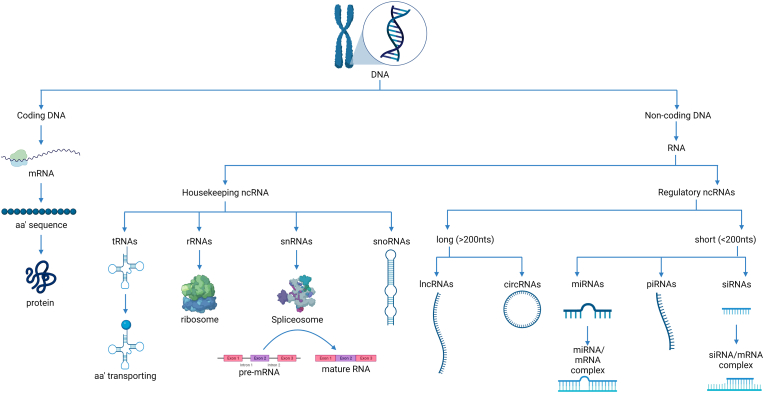
Classification of human genome according to coding potential. This figure represents a classification of human genome (DNA) where DNA includes coding sequences that are transcribed and translated into proteins and non-coding sequences that are only transcribed into RNA. Those RNA molecules are known as non-coding RNAs (ncRNAs) as they lose their coding capacity and to be translated into proteins. ncRNAs are further classified according to its function into housekeeping ncRNAs and regulatory RNAs.

For a long period of time, it was thought that most DNA was coding material that gets translated into protein [1]. However, more than 98% of the human genome was found to be non-coding for proteins while the rest 1-2% encodes ≈21,000 distinct proteins [1]. Lately, the non-coding part of the genome, known as non-coding RNAs (ncRNAs) is classified by function into two categories; housekeeping ncRNAs and regulatory RNAs as presented (Fig. 1) [2]. Housekeeping ncRNAs include transfer RNAs (tRNAs), ribosomal RNAs (rRNAs), small nuclear RNAs (snRNAs), and small nucleolar RNAs (snoRNAs), whereas the regulatory ncRNAs are sub-grouped by length into long ncRNAs (lncRNAs) (>200 nt), and short ncRNAs (<200 nt).

A new subclass of the lncRNAs is the circular RNAs (circRNAs) which have become a matter of interest, due to their rising therapeutic role, high stability profile, high diagnostic and prognostic values, and finally their major contribution to cellular proliferation and the malignant transformation process [3,4].

**Fig. 2:**
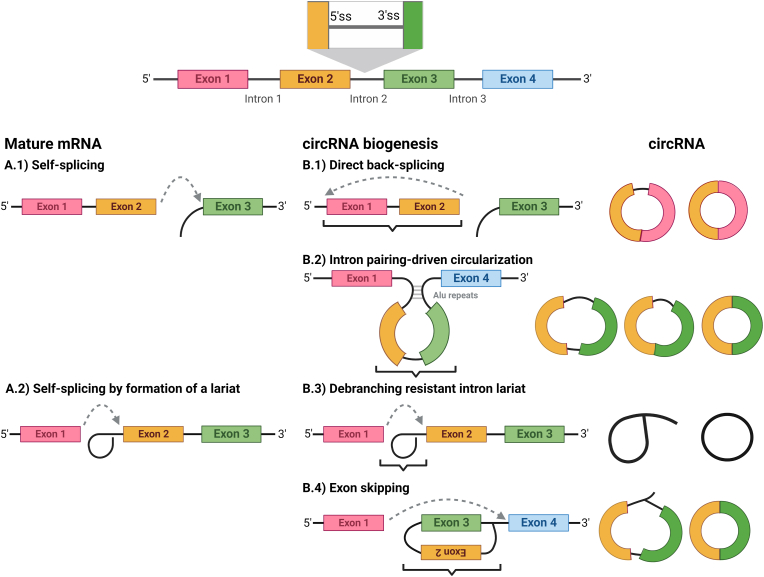
CircRNAs Biogenesis. This is a representative figure showing mature mRNA splicing and corresponding canonical biogenesis of circRNAs which might include B.1) direct back splicing. B.2) Intron pairing driven circularization, B.3) debranching resistant intron lariat and B.4) lariat-driven circularization (exon skipping).

Since most of the eukaryotic genes are interrupted by introns, these introns are removed before protein translation [5]. The transcribed pre-mRNAs undergo certain splicing by the spliceosomes, which are cellular machinery that are able to remove introns to join exons together forming mature RNAs (Fig. 2A) [6]. Hence, splicing can intentionally result in various RNA isoforms since it is highly regulated [7], leading to RNA molecules of different functions, locations and of divergent regulatory roles such as circRNAs [8].

Recently, circRNAs have been casted as novel promising regulators in the field of oncology [9]. It has been reported that circRNAs could behave differently in diverse malignant contexts, in which they could act either as a tumor promotor (oncogenic), or a tumor suppressor circRNA [3,10].

It is notable that unraveling the role of circRNAs in cancer is still a virgin field that needs further investigation [4,11]. Until now, several research groups are focusing on exploring the potential roles of different circRNAs in various solid and humoral malignancies. Yet, the field can still accommodate several research groups to explore the promising role of circRNAs as diagnostic, prognostic biomarkers and/or therapeutic targets or tools in the field of oncology. In the current review, the authors will try to shed the light onto the potential role of circRNAs in breast cancer (BC), which is considered one of the most heterogenous and dominating cancers among females.

## 2 What are CircRNAs?

CircRNAs are generated by special types of splicing and they are mainly found in the cytoplasm [12]. The term “circRNA” was introduced by Sanger et al. in 1976, when an infectious single-stranded covalently closed RNA molecules in plant viroid was characterized [13]. Yet, in the early 2010s, circRNAs were identified in human cells to be of significant abundance [14,15].

Similar to other lncRNAs, circRNAs can serve as miRNA sponges, protein scaffolds, protein decoys and can even have a translational function [16]. However, unlike lncRNAs, most circRNAs get spliced from coding pre-mRNAs [17]. Additionally, due to them lacking 3′ and 5’ free ends and existing as continuous loops, they are highly resistant to degradation by RNAses [18].

### 2.1 Biogenesis of circRNA

*In-vivo* circRNAs are usually originated from protein-coding sequences [19]. The exact mechanisms by which they are produced are shown in Fig. 2 and include I) direct back-splicing [20], II) intron-pairing-driven circularization [21], III) debranching resistant intron lariat [22], and IV) lariat-driven circularization (exon skipping) [23,24]. Typically, pre-mRNAs are spliced by different mechanisms to produce the mature mRNAs, which consist of exons only and are later translated into amino acid sequences [25].

One of the mechanisms by which pre-mRNAs are being regularly spliced is the self-splicing mechanism (Fig. 2A1) [26]. At the end of this process, the free OH′ group at the 5′ splice site (ss) is ligated to the following downstream 3'ss forming a 3′-5′ covalent phosphodiester bond between two consecutive exons [26]. However, when the free OH′ at 5'ss is being peculiarly joined to an upstream 3'ss (in the back), this leads to formation of circRNA that includes exonic parts in its sequence (Fig. 2B1) [20].

Occasionally, if the pre-mRNAs contain two non-adjacent introns containing complementary *Arthrobacter luteus* (ALU) repeats [27], these repeats hybridize/(pair) together bringing flanking introns to become in close proximity, forming a conformational structure that facilitates back splicing of the pre-mRNA (Fig. 2B2) [28,29].

Moreover, mature mRNAs could be produced by different self-splicing mechanisms by which the 5'ss forms a loop-like structure called “lariat” within the same intron to be excised (Fig. 2A2) [30]. The excised intron is usually de-branched by a certain enzyme that was identified in 1985 [31]. However, if the lariat fail to de-branch, an intronic circRNAs may be formed (Fig. 2B3) [22]. However, in Lariat-driven circRNA biogenesis model, which is also called “exon-skipping”, the lariat is extended to form a loop with another downstream-intron, skipping some exons inside the loop structure (Fig. 2B4) [23]. Then, the resulting circRNA could be processed by canonical splicing or not [19].

CircRNAs could obtain sequence of exons only (exonic circRNAs), introns only (intronic circRNAs), or both (exon-intron circRNAs) [32]. The formation of covalently exonic circRNAs occurs when a 3′ end of an exon (5'ss) is joined to a 5′ end of the same exon (single-exon circRNA), or an upstream exon (multiple-exon circRNA), forming a closed RNA loop. However, single-exon circRNA showed longer length than average exon length [33]. Nevertheless, multiple-exon circRNAs which lack intronic sequence is more frequent form of circRNAs [34].

### 2.2 CircRNAs nomenclature

Unfortunately, until now there is no consensus agreement that has been established concerning the nomenclature of circRNAs. There are various nomenclature systems used leading to high level of confusion between similar studies [35]. Despite the great efforts done by different research groups for creating central databases compiling all circRNA data, yet the information is not complete and inconsistent creating isolated islands each of them is using their own language and their own nomenclature system to annotate the circRNAs [36].

Currently, there are 4 available resources that could be considered as databases for circRNAs: circBase [37], circBank [38], CIRCpedia [39], and circAtlas [40]. CircBase is the original database and their proposed nomenclature system was based on the species and some numerical codes added by the database. Later, circBank and circAtlas started to provide a more user-friendly annotation for circRNAs where circRNAs names start to include the gene symbol of the transcriptional unit responsible for its generation based on the genomic coordinate references from UCSC resources [41]. However, CIRCpedia started to use a specific denomination for each circRNA, including the original species and an internal number without the reference to the source gene making it totally different from the nomenclature originally proposed by circBase [37].

The problem is not only that the same circRNA might have several names that are nearly similar but differ in suffix-numerical codes such as CircMTO1 (hsa_circ_0007874, hg19: chr6:74175931–74,176,329) which was found to have 11 different names reported until now. Yet, the real disaster that it was reported that some circRNAs that are nearly identical in names and with the same index sometimes correspond to different circRNAs.

Accordingly, such lack of standardization in such virgin field prevents the required validation of experimental results and the detailed understanding about circRNAs' functional roles and thus hinders the deep knowledge required to be understood about circRNAs at such stage.

### 2.3 CircRNAs interactions with other molecules

#### 2.3.1 CircRNAs-ncRNAs interactions: an endogenous sponge/decoy to microRNAs (miRNAs)

A subclass of the short ncRNAs is microRNAs (miRNAs). miRNAs have an average length of 22 nt and are mostly intragenic; made from introns and few exons of protein coding genes [42].

In fact, miRNAs have been found to transcriptionally regulate the expression of almost 60% of the whole human genome. They are found intracellularly and extracellularly where they can serve as biomarkers for diseases [43,44], malignancies [45], and as a guide for cell-cell interactions [46]. Aberrant expression levels of miRNAs can lead to several malignancies [47]. On the other hand, circRNAs have multiple binding sites for miRNAs [48]. By binding to the miRNAs, circRNAs “sponge” them and prevent them from performing their previously mentioned functions, thus hindering their regulatory functions [32]. Such “sponging” effect could express remarkable impacts on cellular proliferation and cancer progression [32,49]. Using bioinformatics, those miRNA-binding sites can be predicted using web tools such as Circular RNA Interactome (CircInteractome: https://circinteractome.nia.nih.gov/) [50,51]. For instance, circRNF20 harbor miR-487a, acting as miRNA sponge in BC [49]. In non-oncological context, it has also found that circRNA CCDC66 (circCCDC66) acts as a sponge for miR342-3p in vascular smooth muscle cells and ranked as a novel circRNA playing a central role in abdominal aortic aneurysm pathogenesis [52].

### 2.4 CircRNAs-protein interactions

#### 2.4.1 Protein sponges/decoys

By the few protein-binging sites that circRNAs harbor, they could also bind to proteins and sequester them, acting like an antagonist to hinder their physiological function [53]. One of the most common protein classes that bind to circRNA is RNA-binding proteins (RBP). For instance, circ-TNPO3 inhibits gastric cancer (GC) metastasis by decoying insulin-like growth factor 2 binding protein 3 (IGF2BP3) protein [54]. When circ-TNPO3 sequesters IGF2BP3, the expression of MYC and its target SNAIL was suppressed, decreasing the proliferation and metastasis capacity of GC cells [54]. Nonetheless, it was also reported that in colorectal cancer cell lines, circ-SIRT1 bind to the eukaryotic translation initiation factor 4A3 (EIF4A3), hence blocking its inhibitory effect on epithelial mesenchymal transition and promoting the proliferation and invasion of colorectal cancer cell lines [55].

CircRNAs can also serve as protein decoys by binding to cellular proteins and altering their regular physiological function [56,57]. In NSCLC, circ_0000079 (CiR79) sequesters Fragile X-Related 1 (FXR1) protein hindering its complexation with PRKCI, consequently, inhibiting the induction of this complex for cell proliferation and invasion as shown in Table 1 [58]. In addition, ciR79-FXR1 interaction resulted in a decrease in the SNAIL protein levels which is an essential gene for cancer cell growth [58].

**Table 1:** Different functional mode of actions of CircRNAs.

Function	CircRNA	Biological functions	Interacting miRNA/protein	Disease/Cell type	References
miRNA sponge	CDR1 (CiRS-7)	Induce the expression of Ubiquitin protein ligase A (UBE2A)	miR-7	Alzheimer's disease	[69]
	Cir-ITCH	Regulates the expression of ITCH	miR-7	Colorectal cancer	[70]
			miR-17		
			miR-214		
	Circ-LARP4	Regulates the expression of LATS1	miR-424	Gastric cancer	[71]
	Circ-TCF25	Regulates the expression of CDK6	miR-103a-3p	Bladder cancer	[72]
			miR-107		
	Circ-CER	Regulates the expression of MMP13	miR-136	Osteoarthritis	[73]
	Circ-PVT1	Regulates the expression of E2F2	miR-125	Gastric cancer	[74]
	Circ-001564	Unknown	miR-29c-3p	Osteosarcoma	[75]
	Circ-ZNF609	Regulates the expression of ATK3	miR-150-5p	Hirschsprung disease	[76]
	Circ-VMA21	Regulates the expression of XIAP	miR-200c	Intervertebral disc degeneration	[77]
	circMTO1	Regulates the expression of P21	miR-9	Hepatocellular carcinoma	[78]
	circ_0005986	Regulates the expression of Notch 1	miR-129b	Hepatocellular carcinoma	[79]
	circHIPK3	Regulates the expression of IGF-1 and Aquaporin 3	miR-558	Bladder cancer Hepatocellular carcinoma	[80,81]
			miR-124		
			miR-379		
	circACTA2	Regulates the expression of α-SMA	miR-548f-5p	Vascular smooth muscle cells	[82]
Protein Sponge/Decoy	Circ-TNPO3	Suppresses metastasis by regulating MYC and SNAIL	IGF2BP3	Gastric cancer	[54]
	Circ_0000079	Inhibited cell proliferation and invasion	FXR1	NSCLC	[58]
Protein Scaffold	Circ-NDUFB2	Inhibits growth and metastasis	TRIM25	NSCLC	[62]
			IGF2BPs		
	Circ-PDE4B	Inhibit cartilage degradation	RIC8A and MID1	Chondrocyte cell	[63]
	Circ-DCUN1D4	Suppresses metastasis	HuR	Lung adenocarcinoma	[64]
Protein Recruitment	Circ-MRPS35	Tumor suppressor	KAT7	Gastric cancer	[59]
	Circ-SIRT1	Promotes cell Proliferation	eIF4A3	Colorectal Cancer	[55]
Translatable circRNAs	Circ-EIF3J	Enhance RNA Polymerase II activity	U1	HEK293 and HeLa cells	[68]
	Circ-PAIP2	Enhance RNA Polymerase II activity	U1	HEK293 and HeLa cells	[68]

#### 2.4.2 Protein recruitment

CircRNAs could also guide proteins to certain cellular locations in a processes called protein recruitment [57]. It was recently reported that circMRPS35 could potentially repress GC through *in-vitro* and *ex-vivo* analysis, where it was found to act as a modular scaffold to recruit histone acetyltransferase KAT7 to the promoters of *FOXO1* and *FOXO3a* genes. This will elicit acetylation of H4K5 in their promoters, and thus govern histone modification [59]. From a non-oncological perspective, it was found that CircCwc27could directly bind to purine-rich element-binding protein A (Pur-α), increase the retention of cytoplasmic Pur-α, and suppress Pur-α recruitment to the promoters of a cluster of Alzheimer related genes, such as dopamine receptor D1, amyloid precursor protein, regulatory inhibitor subunit 1B, protein phosphatase 1, neurotrophic tyrosine kinase receptor type 1, and LIM homeobox 8 [60].

### 2.5 Protein scaffolds

CircRNAs have different binding sites for proteins [57]. Thus, they can help in protein scaffolding and protein-complex stabilization by facilitating the contact between different proteins [57]. Besides, scaffold formation could initiate nuclear or cytoplamic translocation of the protein through the nuclear pores [61]. For example, circNDUFB2 was found to facilitate the degradation of IGF2BPs by forming a ternary complex between TRIM25 and IGF2BPs, to enhance their interaction [62]. Consequently, IGF2BPs is ubiquitinated and degraded decreasing tumor progression [62]. Furthermore, in human cartilage samples, circPDE4B was found to act as a scaffold for *RIC8A* and *MID*_*1*_ promoting their association, and resulting in proteasomal degradation of *RIC8A* and preventing cartilage degradation [63]. Moreover, circDCUN1D4 in lung adenocarcinoma formed a complex with the RNA-binding protein, HuR protein, promoting its cytoplasmic translocation, and suppressing the metastatic potential of cancerous cells [64].

### 2.6 Translatable circRNAs

Although circRNAs are classified as lncRNAs, they can code for some short peptides [65,66]. The coding circRNAs contain an internal ribosomal entry site (IRES), which allows the translation of some coding sequences within the circRNA [61]. Furthermore, start codon sequence of mRNA was found to be included in many circRNAs molecules [67]. However, the translation of circRNAs usually occurs in a Cap-independent manner since circRNAs do not include 5'cap sequences [65]. Therefore, circRNA translation is more resistant to translation-regulatory proteins, i.e., 4E-BP protein [65].

### 2.7 Miscellaneous functions

It was also discovered that some exon-intron (EI) circRNAs can regulate their own gene transcription [56,68]. For instance, circEIF3J and circPAIP2, as summarized in Table 1 are nuclear circRNAs that can interact with U1; small nuclear ribonucleoproteins (snRNPs) and, hence, enhance RNA polymerase II activity. This activity in turn increases their parental gene transcription [68]. Nevertheless, circRNAs could exert hybrid function such as what circDCUN1D4 does in lung adenocarcinoma [64]. Beside the aforementioned function in translocating HuR protein, circDCUN1D4 also promotes the interaction between HuR protein and thioredoxin-interacting protein (TXNIP) mRNA by acting as a scaffold for them forming a RNA-protein ternary complex showing a mix of different functions of circRNAs typical functions [64]. However, the functional characterization of circRNAs is still in immense need for further investigation to achieve better understanding [50].

## 3 Why breast cancer?

### 3.1 Dominancy of breast cancer among females

BC has recently surpassed lung cancer as the most commonly diagnosed cancer worldwide [83]. In 2020, almost 2.3 million new BC cases were diagnosed, referring to the fact that approximately 1 in every 8 cancer cases diagnosed in 2020 was BC [83]. At the same time, about 658,000 BC-related deaths were reported in 2020 [83]. BC is a highly complex disease. It might be referred to as several diseases that occur in one organ [84,85]. Although it affects only one anatomic site, it is a heterogeneous disease with great phenotypical variability [86].

Several laboratory techniques have been utilized such as immunohistochemistry [87] and gene expression profiling, in order to classify the various subtypes of BC [88]. To date, accumulating evidence supports the fact that this disparity in biological subtypes is concomitant with distinctions in treatment response and disease-specific outcomes [89]. Several factors such as tumor morphology and grade, tumor size, lymph node metastases, and expression of estrogen receptor (ER), progesterone receptor (PR) and human epidermal growth factor receptor-2 (HER-2/neu) are currently known as important parameters essential for treatment tailoring. However, there is a great necessity to further enhance the understanding of prognostic and diagnostic biomarkers that will streamline the process of precision medicine [90].

Resistance of cancer cells to conventional therapeutic approaches such as chemotherapeutic drugs has necessitated the direction toward the personalized and targeted therapies [91]. Even the responsiveness for targeted therapies and immunotherapy such as immune checkpoint blockades (ICBs) is only effective in a subset of cancer patients [92]. To increase the survival rate in BC patients, novel therapeutic targets should be discovered and investigated to help in uncovering the crosstalk among oncogenic signaling pathways [92,93].

In the second part of this review, the authors will focus on the complexity of cellular and molecular heterogeneity among BC tumors, focusing on the novel role of circRNAs as a new layer of complexity evading BC heterogeneity. Also, this review will crystalize the potential role of circRNAs in BC and its utilization capacity to act as stable non-invasive biomarkers and/or therapeutic tool in BC precision medicine.

## 4 Heterogeneous nature of BC tumors

### 4.1 Inter-tumor and intra-tumor heterogeneity in BC

BC molecular heterogeneity is on both levels intra-tumoral and inter-tumoral. Inter-tumor heterogeneity is noted as the presence of various types of BC with different molecular phenotypes among different individuals, whilst intra-tumor heterogeneity is due to the existence of heterogeneous cell populations within an individual tumor mass [94].

Inter-tumor heterogeneity leads to the existence of different BC subtypes which are categorized by their molecular profiles, morphology, and expression of specific biomarkers (*ER*, *PR* and *HER2*) [95]. This results in BC variability with unique biological behaviors and diverse drug resistance and clinical outcomes [96]. On the contrary, intra-tumoral heterogeneity is found within the tumors of the same type [94]. It is the tumor's ability to acclimatize and adjust to new tumor microenvironment (TME) settings. This new setting may be caused by chemotherapy, radiotherapy, severe ischemia, or a decline in nutrients [97]. Consequently, the tumor specimen taken during a biopsy could not be a representative of the entire real tumor composition, because the tumor may comprise phenotypically different cancer cell populations with different properties and resistance to drugs [98]. In fact, genetic mutations and/or epigenetic modifications are the source of intra-tumor heterogeneity, which explains why the same cell types have dissimilar phenotypic variants [97]. Moreover, this gives a reason why TME components (e.g., different populations of cancer fibroblast or stromal heterogeneity), immune system infiltration, or even the dysregulation of the extracellular matrix, may result in variability in tumor composition [98]. Fascinatingly, the histological and genetic profiles of tumors in patients remain unaltered over their lifetime despite their heterogeneity, whether they metastasized, or remained localized, and even at the end-stage disease [99].

One of the ways to decode the BC heterogeneity may be the identification of different cell phenotypes, cell density, or their localization in the tumor. Heterogeneous cells usually have different molecular pictures within individual tumors [98]. However, heterogeneity could also be predicted by the BC grade [95]. Since the grade of BC is considered as an important prognostic factor, it is incorporated in tools such as Nottingham Prognostic Index (NPI) as well as PrognosTILs [100].

### 4.2 Heterogeneity on the receptors level

BC cells usually have molecular markers such as ER or PR hormone receptors (*HR*) expression, and *ERBB2* gene amplification, formerly known as *HER2* receptor [101]. Based on the most recent European Society of Medical Oncology (ESMO) guidelines, BC can be subdivided into four molecular subtypes: 1) luminal A, in which HR are expressed while the *HER2* is absent (*HR*^*+*^*/HER2*^*−*^); 2) luminal B, in which *ER* is positive while *HER2* may or may not be expressed (*ER*^*+*^*/HER2*^*−/+*^); 3) *HER2* enriched, in which (*HR*^*−*^*/HER2*^*+*^); and 4) basal-like subtype, which is also known as triple-negative BC (TNBC), tumor cells do not possess any of the 3 standard molecular markers (*HR*^*−*^*/HER2*^*−*^) [84,102,103].

The markers of heterogeneity are defined as the difference in the expression of these receptors [95]. For instance, *HER2*^*+*^ tumors are aggressive tumors yet are usually treated with anti-HER2 therapy while ER-positive tumors are mostly well-differentiated, less aggressive, and linked to better post-surgery results [104]. On the other hand, TNBC is a highly heterogeneous cancer in regard to its molecular and phenotypical features [105]. TNBC is the most aggressive and most likely to recur out of those four groups [106]. Based on such sub-classification, the patient's treatment protocol is decided [107], taking into consideration the aforementioned histological and anatomic cancer stage [108]. Collectively, some of BC subtypes have better prognosis and response to treatment, while other subtypes are notoriously aggressive, with a poor prognosis and reaction to treatment that again reflects the heterogeneity of BC tumors [109].

### 4.3 Heterogeneity on the genetic level

Scientists have exploited genetic heterogeneity to be used as a risk factor used to identify the risk of BC occurrence and even helps in predicting its possible subtype. Mutations in *BRCA* genes are among the most clearly established risk factors for BC [110,111]. About 12% of BC patients under 40 years of age were found to have *BRCA* gene mutations [112]. *BRCA1* and *BRCA2* are considered as BC susceptibility genes [110]. They are tumor-suppressor genes involved in DNA repair and mutations in these genes confer a 45–65% risk of BC by the age of 70 years [111]. TNBC tumors are identified by the high occurrence of germ line *BRCA1/2* mutations, almost twice its frequency in BC patients [112].

### 4.4 Heterogeneity on the epigenetic level

Although genetic changes have been recognized long time ago as a key player in BC development, heterogeneity in epigenetic profile in BC tissues compared to healthy tissues has directed studies to focus on this level of cell regulation as to be a potential initiator for tumor progression [113]. Epigenetic changes such as chromatin remodeling and alteration in expression levels of ncRNAs, all could affect gene expression. Due to availability of methodological approaches, DNA hypermethylation and changes in ncRNAs profile have been studied more intensively in literature, compared to studies on histone modifications in cancer patients [114]. Yet, fluctuation in ncRNAs expression levels has not only correlated to BC hallmarks, but also has been linked to chemotherapeutic resistance [115]. Studies on miRNAs and lately lncRNAs have remarkably increased in the last decades, showing aberrant expression that could be exploited as potential diagnostic and prognostic markers for all cancer types generally and for BC in particular [116]. On the other hand, circRNAs, which are relatively more recent and less investigated compared to miRNAs have shown superior characteristics attributing them a higher potential for clinical application [117]. Stability and conservation of circRNAs which can be secreted from cells and tissue into the serum could be used as a biomarkers to help in disease detection and for treatment response follow-up [14].

## 5 CircRNAs: Revolution Era in the prognosis and diagnosis of BC

The prognostic and diagnostic biomarkers for different malignancies have been investigated for centuries, however, the survival rate in patients have not become promising enough [118]. As mentioned in the previous section, cancer heterogeneity represents a great challenge in treatment strategies [119,120]. Albeit that, the treatment protocol should be further studied to reach better clinical outcomes [118]. CircRNAs-based biomarkers present an emerging and promising pivotal role in tumor detection, treatment, likelihood of recurrence, and metastasis property prediction [50,121]. Therefore, previous research articles have tackled circRNAs role in tumorigenesis generally and in BC particularly (Table 2) [3,[122], [123], [124], [125], [126], [127], [128], [129]].

**Table 2:** CircRNAs evading the field of oncology.

Functional Activity	Cancer Type	circRNA	References
Tumor-Enhancer	Breast cancer	Circ-0044234	[133]
		Circ-NOTCH3	[134]
		Circ-TP63	[135]
		Circ-0000284 (circHIPK3)	[136,137]
		Circ-0007766 (circ-ERBB2)	[138,139]
		Circ-0019853 (circ-PDCD11)	[140]
		Circ-0084100 (circIKBKB)	[141]
		Circ-0008673	[142]
		Circ-NOLC1	[143]
		Circ-0055478 (circPTCD3)	[144]
		Circ-0000073 (circOMA1)	[145]
		Circ-0001944 (circBCBM1)	[146]
		Circ-0103552	[147]
		Circ-0084927	[148]
		Circ-RHOT1	[149]
		Circ-0092338 (circNINL)	[150]
		Circ-CNOT2	[151]
		Circ-0048764	[152]
		Circ-0000515 (circRPPH1)	[153,154]
		Circ-0000515	[155]
		Circ-DNMT1	[156]
		Circ-PGAP3	[157]
		Circ-BACH2	[158]
		Circ-ABCB10	[159,160]
		Circ-0011946	[161]
		Circ-gfra1	[162]
		Circ-0001982	[163,164]
		Circ-0005230	[118]
		Circ-IRAK3	[139,165]
		Circ-0008039 (circFOXO3)	[166]
	Glioma	Circ-TTBK2	[167]
		Circ-NT5E	[168]
	Hepatocellular carcinoma	Circ-ZFR	[169]
		Circ-FBLIM1	[170]
		Circ-HIPK3	[81]
	Cervical Cancer	Circ-000284	[171]
		Circ-0023404	[172]
	Lung cancer	Circ-102231	[173]
		Circ-PRKCI	[174]
		Circ- MAN2B2	[175]
		Circ-0013958	[176]
	NSCLC	Circ-ZFR	[177]
		Circ-0007385	[[178], [179], [180]]
		Circ-100146	[181]
		Circ-FOXO3	[182]
		Cirs-7 (CDR1as)	[183,184]
	Colorectal cancer (CRC)	Circ-CCDC66	[185]
		Circ-HIPK3	[186]
		Circ-0000069	[187]
		Circ-001569	[188]
	Oesophageal squamous cell carcinoma	Cirs-7 (CDR1as)	[189]
		Circ-HIPK3	[190]
	Gastric Cancer	Circ-0047905	[191]
	Oral Squamous Cell Carcinoma (OSCC)	Circ-DOCK1	[192]
	Clear cell renal carcinoma (ccRCC)	Circ-ATP2B1	[190]
	Papillary Thyroid Cancer	Circ-ZFR	[193]
	Melanoma	Circ-0084043 (CircADAM9)	[194]
	Prostate Cancer (PC-a)	Circ-MYLK	[195]
	Gall Bladder Cancer	Circ-HIPK3	[196]
	Head and neck squamous cell carcinoma	Circ-PVT1	[197]
Tumor-suppressor	Breast cancer	Circ-0006220	[198]
		Circ-0120472 (circCCDC85A)	[199]
		Circ-NR3C2	[200]
		Circ-0000442	[201]
		Circ-tada2a-E6	[202]
		Circ-000911	[203]
		Circ-0072309	[204]
		Circ-ASS1	[205]
	Glioblastoma	Circ-FBXW7	[206]
		Circ-SHPRH	[207,208]
		Circ-0001445 (Circ-SMARCA5)	[209]
		Circ-0001946	[210]
	Hepatocellular carcinoma	Circ-C3P1	[211]
		Circ-MTO1	[78]
		Circ-0001445 (Circ-SMARCA5)	[212]
		Circ-ZKSCAN1	[213]
		Circ-ADAMTS13	[214]
		Circ-ADAMTS14	[215]
		Circ-0079299	[216]
		Circ-SMAD2	[217]
	Gastric Cancer	Circ-TNPO3	[54]
		Circ-YAP1	[218]
		Circ-0000993	[219]
		Circ-PSMC3	[220]
		Circ-FAT1(e2)	[221]
		Circ-0000096	[222]
		Circ-larp4	[71]
		Circ-ZFR	[223]
	Lung cancer	Circ-dcun1d4	[64]
		Circ-100395	[224]
		Circ-0006916	[225]
		Circ-NOL10	[226]
	NSCLC	Circ-0000079 (Cir79)	[58]
		Circ_0008305 (CircPTK2)	[227]
		Circ-0001649	[228]
		Circ- ITCH	[229]
		Circ-0043256 (Circacaca)	[230]
	Colorectal cancer (CRC)	Circ-ITGA7	[231]
		Circ-0014717	[232]
		Circ-ITCH	[70]
	Bladder Cancer	Circ-FNDC3B	[233]
		Circ-LPAR1	[234]
		Cirs-7 (CDR1as)	[235]
		Circ-HIPK3	[236]
		Circ-ITCH	[237]
	Papillary thyroid cancer	Circ-ITCH	[238]
	Ovarian cancer	Circ-ITCH	[239]
	Osteosarcoma	Circ-0002052 (Circpappa)	[240]
	Oral squamous cell carcinoma	Circ-0008309	[241]
	Cholangiocarcinoma	Circ-0001649	[242]

CircRNAs-based diagnostic and prognostic biomarkers are recently used in BC detection, grading, and follow-up since they show BC-tissue specificity and could discriminate cancerous cells from adjacent healthy tissue. Besides, circRNAs may help in differentiation between BC subtypes, giving a fast hint about the treatment protocol to be followed with the patient, and thus, participating in better outcomes. Indeed, prognostic circRNAs themselves could be targeted for therapy, since silencing their expression or exploiting them as therapeutic targets would contribute in enhancing tumor prognosis.

Nonetheless, based on the current understanding of the circRNAs nature, it makes them perfect match for a qualified diagnostic and/or prognostic biomarker. CircRNAs have 4 characteristics that ranks it as a potential biomarker for several oncological diseases including BC. CircRNAs are **1) Stable:** this is due to the covalently closed loop structure, therefore making it highly resistant to exonuclease RNase R and more stable and have longer half-live in plasma than any other linear lncRNA [130,131]. **2) Universal:** circRNAs have been reported to be the most universal molecule present within the human cell and usually the circRNA is more abundant that its linear form [132]. **3) Specific**: circRNAs are reported to be expressed in a tissue-specific manner and in a developmental stage-specific manner which makes them perfect candidates for promising oncological biomarkers [131]. **4) Conservative:** circRNAs are evolutionally conserved in different species such as murine models and human beings [131]. In the following section of the review, the authors will discuss the newly discovered prognostic and diagnostics circRNAs used in BC.

### 5.1 CircRNAs: promising diagnostic biomarkers in BC

The circRNA hsa_circ_0000284 (circHIPK3) was found to be overexpressed in BC tissues compared to its normal counterparts ranking is as potential diagnostic circRNA for BC patients. However, this has further investigations to be validated. Functionally, CircHIPK3 was found to act as a sponge for miR-326. High level circHIPK3 has been associated with poor prognosis. CircHIPK3 was reported to induce cellular proliferation, migration, and invasion ability of the BC cells. In addition, circHIPK3 was found to inhibit the apoptosis of the BC cells. However, this effect is reversed when circHIPK3 was knocked down, highlighting its potential as promising therapeutic target as well [136].

Circ_0008673 was also found to be overexpressed in BC cells. On the molecular level, Circ_0008673 was found to induce CFL2 expression level by sponging miR-153-3p. Due to the high levels of circ_0008673 in BC tissues, elevated levels of CFL2, which is an actin-binding protein, was observed in the BC tissues which leads to increase cell proliferation, migration and invasion capacities of BC cells and decreasing their apoptosis [142]. Collectively, this highlights circHIPK3 and Circ_0008673 as oncogenic mediators and potential diagnostic circRNAs in BC patients.

**Fig. 3:**
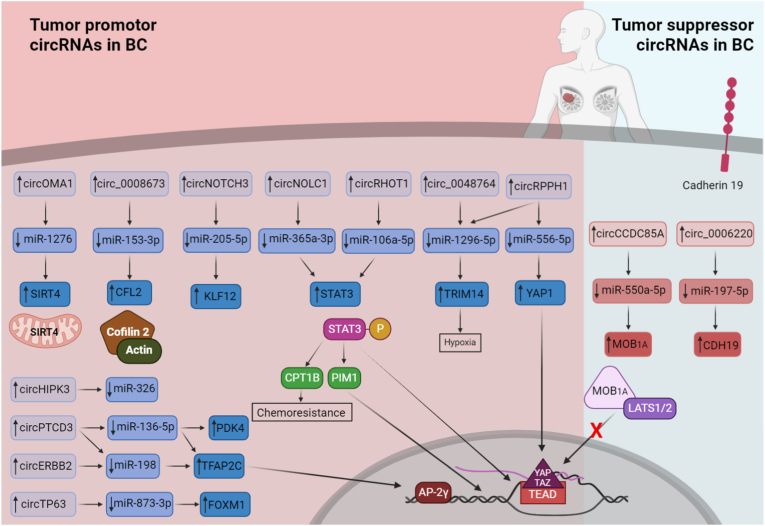
Different tumor suppressor and tumor promotor circRNAs in BC. This figure represents an array of tumor suppressor circRNAs and tumor promotor circRNAs with their respective target and their determining role in breast cancer.

circNOLC1 was also reported to participate in promotion of BC via regulating miR-365a-3p/STAT3 axis as shown in Fig. 3. BC tissues and cell lines have expressed higher level of circNOLC1 compared to normal tissues, eliciting high expression level of STAT3. However, propofol treatment has repressed the expression of both circNOLC1 and STAT3, decreasing BC cells viability [143]. Yet, additional studies have shown that the activation of STAT3 signaling pathway activates CPT1B, which is a cellular molecule that contributes to chemoresistance as represented in Fig. 3 [243]. However, circRHOT1-activated STAT3 is intermediated by sponging miR-106a-5p (Fig. 3) [149]. In a study by Zhang *et al*, the increase of STAT3 levels via circRHOT1/miR-106a-5p/STAT3 axis has resulted in ferroptosis attenuation in BC cells. However, knockdown of circRHOT1 has retained cell apoptosis ability, increased the levels of ROS and iron in BC cells [149]. Intriguingly, a proto-oncogene *PIM1,* which is responsible for cell invasion and epithelial-mesenchymal transition (EMT) promotion through upregulating the expression of EMT-related genes in BC, is found to be regulated by the phosphorylated STAT3 as shown in Fig. 3 [243].

hsa_circRNA_0000073 (circOMA1) has been found to be upregulated in BC tissues (Fig. 3). CircOMA1 fosters BC progression by sponging miR-1276, leading to elevation of the mitochondrial matrix protein Sirtuin 4 (SIRT4). SIRT4 has a controversial role in cancer development. It showed anti-tumor activity in lung and colorectal cancer cells, yet, it could act as oncogene in esophageal cancer and BC tissues [145].

Correspondingly, hsa_circ_0000515 (circRPPH1) have been reported previously to participate in TNBC development and progression by regulating miR-556-5p/YAP1 axis [153]. Yet, similar to circ_0048764 [152], circRPPH1 was recently reported to regulate miR-1296-5p/TRIM14 axis in TNBC tissue promoting hypoxia-associated TNBC progression [154].

For hsa_circ_0007766 (circ-ERBB2), it was found to participate in BC development by regulating miR-136-5p/PDK4, miR-136-5p/TFAP2C, or miR-198/TFAP2C axes [138,139]. Similar to circ-ERBB2, hsa_circ_0055478 (circPTCD3) was also found to act as miR-198 sponge. Elevated levels of circPTCD3 in BC tissues and cell line reverse the antiproliferative activity of miR-198, boosting BC development [144].

Intriguingly, circRNAs could be used as a discriminator to differentiate BC subtypes [[133], [134], [135]]. Although hsa_circ_0044234 is upregulated in BC tissues, it remarkably decreased in the TNBC subtype. Therefore, it could be used to discriminate between TNBC and other BC subtypes (being lower in TNBC) with 83.64% specificity and 72.5% sensitivity. Moreover, the downregulation of hsa_circ_0044234 in TNBC cells was found to lead to overexpression of miR-135b-5p by 4.89 units for each unit-decrease in the levels of hsa_circ_0044234. The increase of miR-135b-5p could also lead to decrease of GATA3 expression, which is needed for homologous recombination DNA repair pathway. Thus, TNBC cells usually suffer from the disturbance of this pathway [133]. A study by Guan *et al* has assured that basal-like BC (BLBC) development and progression involves circ_NOTCH3. Compared to normal breast tissues, BLBC showed higher expression level of circ_NOTCH3 which lead to sponging miR-205-5p and over production of KLF12 protein [134]. Meanwhile, ER^+^ BC showed higher expression levels of circTP63. circTP63 induces cancer progression via upregulating FOXM1 production by sponging miR-873-3p, which is an inhibitor of FOXM1 mRNA translation [135].

Throughout the past year, few tumor-suppressor circRNAs were discovered. For instance, hsa_circ_0006220 has the ability to suppress TNBC proliferation, migration, and invasion ability by sponging miR-197-5p, which is a natural silencer for CDH19 gene expression. Consequently, hsa_circ_0006220 promotes an increase in cadherin-19 protein cellular levels, retaining its role in maintaining intercellular connections (Fig. 3). However, hsa_circ_0006220 was found to be remarkably downregulated in TNBC tissues compared to other BC subtypes [198].

Oncogenic miR-550a-5p has expressed high intracellular levels in BC tissues leading to increase in their proliferation, migration, and invasion ability by hindering MOB1A activity [199]. Induction of hsa_circ_0120472 (circCCDC85A) expression in BC cells and tissues, which was found to be initially down regulated, has reversed the oncogenic properties of miR-550a-5p (Fig. 3) [199]. MOB1A has shown intriguing role in limiting tumor progression as a part of Hippo signaling pathway. Upon phosphorylation by MST1/2 kinases, MOB1A/1B interact with LATS1/2 kinases forming a complex that leads to subsequent auto phosphorylation of LATS [244]. YAP and its paralog TAZ are key transcriptional regulators that are phosphorylated by LATS kinases leading to their sequestration or degradation, hindering their translocation to the nucleus and prohibiting their contribution to gene expression when bind to TEAD transcription factors (Fig. 3) [244].

### 5.2 CircRNAs: promising prognostic biomarkers in BC

**Fig. 4:**
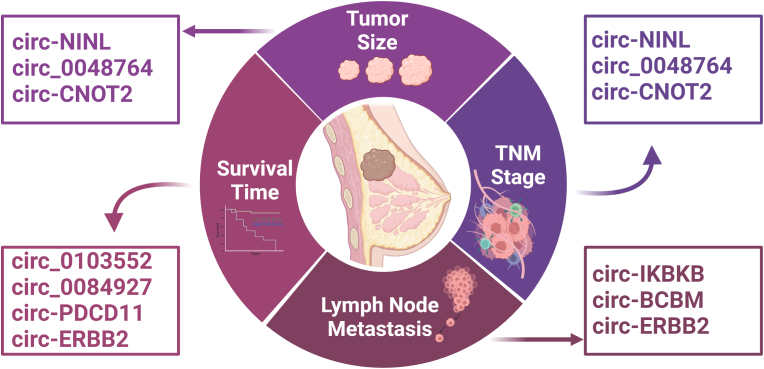
Different promising prognostic circRNAs in BC. This figure represents an array of circRNAs which are correlated to survival time, tumor size, TNM stage, and lymph node metastasis in BC representing promising BC prognostic biomarkers.

Prognostic biomarkers are crucial when it comes to clinical practice. Since circRNAs are stable, conserved, and easily processed molecules that show high tissue specificity, plentiful studies have investigated the potential role of circRNAs as prognostic biomarkers to help in following the clinicopathological states of BC patients [245]. During the past year, number of circRNAs was correlated to patient survival time (Fig. 4). One of BC tissue-specific circRNAs is circ_0103552, it was found to promote BC progression through sponging miR-515-5p, which leads to upregulation of the angiogenic factor CYR61 promoting BC tumorigenesis [147]. BC patients survival time was found to be shorter for patients who displayed high circ_0103552 expression profile [147]. Similarly, circ_0084927 was correlated with BC prognosis and its downregulation was linked to longer survival period. However, on the molecular level, circ_0084927 sponges miR-142-3p, leading to upregulation of ERC1 protein [148]. Likewise, when the oncogene hsa_circ_0019853 (circ-PDCD11) is upregulated in TNBC, it is to be correlated to unfavorable survival and prognosis since circ-PDCD11 sponges miR-432-5p leading to promotion of LDHA expression. Accordingly, the rate of glucose uptake, lactate generation, ATP synthesis and microenvironment acidification was increased, enhancing aerobic glycolysis [140]. [246]. Nevertheless, expression level of circ-ERBB2 has been negatively correlated with poor TNBC survival by contributing in TNBC cells proliferation and invasion. When circ-ERBB2 cytoplasmic levels increases, it sponges miR-136-5p, leading to upregulation of PDK4 and enhancing of Warburg effect [138].

As such, other circRNAs were correlated with larger tumor size, advanced TNM stage, and lymph node metastasis (Fig. 4). Hsa_circ_0092338 (circNINL), circCNOT2 and circ_0048764 were highly correlated with poorer TNM stage and lager tumor size [[150], [151], [152]]. CircNINL has shown elevated expression levels in BC cells compared to normal tissue [150]. It was found to elicit BC tumorigenesis by promoting the transcription of oncogenes which are regulated by β-catenin signaling pathway. This is done by sponging of miR-921 on circNINL leading to upregulation of ADAM9, allowing its interaction with E-cadherin initiating the β-catenin signaling pathway [150]. Likewise, circCNOT2 was found to be upregulated in BC tissues leading to their poor prognosis by promoting the expression of TWIST1, a helix-loop-helix transcription factor, via sponging miR-409-3p [151]. However, circCNOT2 was also linked to low patient's survival rates [151]. Moreover, circ_0048764 partakes in BC tumorigenesis by sponging miR-1296-5p, similar to circRPPH1, leading to elevation of TRIM14 which is known as a cancer-progression promoting factor whose depletion induces apoptosis and results in better cancer prognosis [152].

On the other hand, hsa_circ_0084100 (circIKBKB), hsa_circ_0001944 (circBCBM), and hsa_circ_0007766 (circ-ERBB2) did not only show the potential to predict BC metastatic ability, but also BC intrinsic metastatic property and destination [139,141,146]. A study done by Xu et al., they discovered that circIKBKB overexpression is strongly correlated with BC bone metastasis by NF-κB-mediated mechanism that results in osteoclastogenesis induction. Generally, the synthesis of IκBα, which is a natural NF-κB inhibitor is mediated by activated NF-κB. IκBα bind to N-terminal of NF-κB and hinder its binding to chromatin and export it back to the cytoplasm in a form negative feedback loop. When circIKBKB present in the cytoplasm, it bound to active IKKβ and p65 facilitating their interaction. Thus, instead of formation of p65/IκBα complex and maintaining the negative feedback loop, circIKBKB results in IKKβ-mediated IκBα phosphorylation and promotion of NF-κB pathway which in turn upregulation of many bone-remodeling factors, and thus, induction of osteoclastogenesis and BC bone metastasis [141]. Meanwhile, circBCBM1 expression levels were correlated to BC-brain metastasis in tissues, cell lines and plasma samples. CircBCBM1 sponges miR-125a leading to subsequent increase in BRD4, a member of epigenetic regulator protein family. The elevated BRD4 levels induce MMP9 production via SHH signaling pathway. Secretion of MMP9 typically increased BBB permeability. Therefore, upregulation of BRD4-MMP9 axis facilitated BC-brain metastasis [146]. As such, expression of circ-ERBB2 was linked to lymph node metastasis in ERBB2+/HER2+ BC patients by working as miR-136-5p or miR-198 sponge. Consequently, TFAP2C, also called AP-2γ, is upregulated. TFAP2C is known to be a key mediator in cell cycle and cell apoptosis regulation. When the expression levels of circ-ERBB2 increased, cell proliferation, invasion, and migration was promoted, whereas and cell apoptosis was delayed [139]. In addition, circ-ERBB2 levels were correlated to HER2 receptor status, whether it was present or not [139].

## 6 Conclusion

This review sheds the light onto a fast-growing field of research. It is quite evident that the field of circRNA has witnessed an enormous growing number of publications in few years. CircRNAs have been casted as pivotal players in the tumorigenic process. CircRNAs have been proven to act as miRNA sponges and have a intertwined interaction with several oncogenic proteins thus affecting several cancer hallmarks such as proliferation, migration, invasion, angiogenesis and apoptosis. CircRNAs have been reported to act as promising therapeutic tools/targets in BC. However, circRNAs have not been well studied as prominent players at the cancer-immune synapse in the TME of BC or any other solid malignancy. This underscores the importance of directing future studies on studying the onco-immunogenic role of circRNAs in BC, and its possible involvement in the crosstalk between cancer and effector immune cells at the TME as well. In BC, circRNAs have been discussed as potential diagnostic and prognostic biomarkers. However, this area still needs further experimental validation before its clinical translation.

## 7 Future Recommendations

Notwithstanding the need for further understanding for the physiological and pathological roles of circRNAs, it is evident that they are involved in almost all biological processes. The interpretation of the mechanistic points of all circRNAs associated with BC, either functioning as oncogenes, or as tumor suppressors will be a significant addition to further unlocking minds in the realm of BC. Indeed, this could be achieved by deeply comprehending how heterogeneous malicious TNBC operates and the prospect of targeting circRNAs. All the aforementioned details imply the alarming intricacy and convolution of the BC, and how such a multi-layered disease will need intensive research and ingenuity. Finally, the authors highly appraise the great potential of circRNAs and that they would provide a promising therapeutic tool for this predicament, and that with the unraveling and interpreting the deep sea of circRNAs, which may serve as prognostic, diagnostic, and even therapeutic tools, or molecules to be targeted for cell cycle control. Hence, this could be achieved by advanced analysis and investigation in the field.

## CRediT authorship contribution statement

**Alyaa Dawoud:** Data curation, Formal analysis, Literature review, data analysis, original draft preparation, Tables and Figures preparation. **Zeina Ihab Zakaria:** Data curation, Formal analysis, Literature review, data analysis, original draft preparation. **Hannah Hisham Rashwan:** Data curation, Formal analysis, Literature review, data analysis, original draft preparation. **Maria Braoudaki:** Writing, revising the manuscript critically for important intellectual content and editing the final version of the manuscript. **Rana A. Youness:** Conceptualization, and design of work, writing, revising the manuscript critically for important intellectual content and editing the final version of the manuscript. All authors read, revised and approved the final submitted manuscript.

## Abbreviation List

ALU: Arthrobacter luteus
BL1: basal-like
BL2: basal-like 2
MES: mesenchymal
MSL: mesenchymal stem-like
IM: immunomodulatory
LAR: luminal androgen receptor
BLIA: basal-like immune-activated
BLIS: basal-like immunosuppressed
TME: tumor microenvironment
ICB: immune checkpoint blockades
DNA: Deoxyribonucleic acid
RNA: Ribonucleic acid
rRNA: Ribosomal RNAs
tRNAs: Transfer RNAs
snRNAs: Small nuclear RNAs
snoRNAs: Small nucleolar RNAs
pre-mRNA: Precursor messenger RNA
RNA pol II: RNA polymerase II
DGCR8: DiGeorge Syndrome Critical Region 8
HER2: Human Epidermal growth factor Receptor 2
ERBB2: Erb-b2 receptor tyrosine kinase 2 (ERBB2)
NSCLC: Non-small cell lung cancer
EMT: epithelial-mesenchymal transition
TFAP2C: Target transcription factor activator protein 2C
